# Carbon nanotube/waterborne polyurethane nanocomposites with light-to-heat conversion properties

**DOI:** 10.55730/1300-0527.3555

**Published:** 2023-03-21

**Authors:** Hayriye ÜNAL

**Affiliations:** Sabancı University SUNUM Nanotechnology Research Center, İstanbul, Turkey

**Keywords:** Photothermal coatings, waterborne polyurethane, multiwalled carbon nanotubes, light-activated antibacterial activity, sunlight-activated self-healing

## Abstract

Photothermal materials and coatings which can create temperature elevations under light irradiation can be utilized in various applications requiring remote heating. Here, multiwalled carbon nanotubes (MWNT) were incorporated into waterborne polyurethane (PU) to obtain photothermal coatings with light-to-heat conversion properties. Resulting PU-MWNT coatings were demonstrated to heat up to 80 °C under sunlight irradiation at 2 sunlight density for 18 min. *Pseudomonas aeruginosa* (*P. aeruginosa*) cells attached to surfaces coated with PU-MWNT nanocomposites were killed upon near infrared (NIR) light irradiation at 808 nm for 15 min, whereas the same cells attached to control neat PU-coated surfaces remained alive under the same irradiation conditions. Furthermore, a scratch of 1 cm width on the PU-MWNT coating was shown to be healed under 12 min of sunlight irradiation. The PU-MWNT nanocomposites have strong potential as photothermal coatings, which can be remotely heated with NIR light activation.

## 1. Introduction

Photothermal coatings and materials presenting light-to-heat conversion properties offer important novel functionalities in different applications. Materials that heat up to certain temperatures when remotely irradiated with light are being utilized in various applications ranging from disrupting tumors to physically inactivating microbes and from evaporating water via sunlight to stimulating release from a cargo [1–4]. Photothermal materials convert the absorbed near infrared light to heat via plasmonic localized heat conversion, electron-hole generation, or HOMO-LUMO excitation and lattice vibrations [5]. This photothermal effect resulting in light-activated temperature elevations has been demonstrated in metal nanoparticles, carbon-based semiconductors such as graphene and carbon nanotubes, in conjugated polymers such as polydopamine, polyaniline, and some organic dye molecules [6]. Nanocomposites into which photothermal nanoparticles have been integrated have also been demonstrated to effectively present light-to-heat conversion properties and be utilized in applications, including light-activated photothermal therapy, water evaporation, self-healing, and shape memory [1, 7–11].

Coating materials which can present temperature elevations when irradiated with light can offer important functionalities. The light activated temperature elevations obtained from a coating can be utilized for light-activated killing of bacteria, for the light-activated controlled release of an active agent such as a corrosion inhibitor, or for the remote healing of a defect in the coating. Photothermal coatings have been designed by the deposition of photothermal agents on surfaces via different thin film technologies and utilized for catalysis [12], desalination [13], antiicing [14], antifogging [15], membrane distillation [16], or active-repairing [17]. Incorporation of photothermal agents into polymers resulting in photothermal polymeric nanocomposite coatings, on the other hand, allows obtaining flexible coatings with strong mechanical properties presenting photothermal properties, which can be easily applied on large-scale surfaces. In a limited number of studies, polymeric coatings were imparted with light-to-heat conversion properties by incorporating photothermal fillers in epoxy or polyurethane matrices resulting in coatings with light-activated self-healing properties [17–21].

Waterborne polyurethanes are environmentally friendly polymers with versatile chemistry yielding coatings with tunable thermomechanical properties, which find a multitude of industrial applications [22]. In this study, waterborne polyurethanes have been converted to photothermal nanocomposites by the incorporation of carbon nanotubes as photothermal fillers. Composites of waterborne polyurethane and carbon nanotubes have been reported in the literature before, demonstrating that carbon nanotubes can improve mechanical properties, increase melt viscosity, or introduce different functionalities like gas sensing or electromagnetic interference shielding effect [23–25]. However, carbon nanotubes were not incorporated into waterborne polyurethane matrix to impart photothermal character before. Here, sunlight-activated remote heating properties of the waterborne polyurethane/carbon nanotube composite coatings and their potential in photothermal applications have been studied.

## 2. Materials and methods

### 2.1. Materials

MWNTs with 13–18 nm outer diameter, 3–30 μm length, and 99 wt.% purity were provided by Cheap Tubes Inc. (Cambridgeport, VT, USA). Triton X-100 was purchased from Merck Millipore (Darmstadt, Germany). *P. aeruginosa* (ATTC 27853) cells were purchased from Medimark (France). Nutrient broth (NB) was purchased from Biolife (Milan, Italy). The LIVE/DEAD BacLight bacterial viability kit (L7012) was purchased from Life Technologies (Carlsbad, CA, USA). Anionic, aqueous PU dispersion based on a polyester–polyol was kindly supplied by Punova R&D and Chemicals Inc. (Turkey) with a 32 wt.% solid content.

### 2.2. Preparation of MWNT dispersions

A dispersal solution (10 mL) containing 0.2 mg/mL MWNTs in an aqueous solution of 5 v/v % Triton X-100 was sonicated in an ice-bath with a microprobe (QSonica, Q700) for 20 min with 2 s pulse on and 3 s pulse off time at 50% amplitude and a power of 4–5 W. Aqueous dispersions were centrifuged at 14,000 rpm for 10 min to remove MWNTs that were not dispersed. The black-colored supernatant was pipetted into a clean falcon tube. Concentration of MWNT dispersions were determined by absorbance spectroscopy (Cary 5000 spectrophotometer) using the specific extinction coefficient for MWNTs at 500 nm (ɛ_500_ = 46 mL mg^−1^ cm^−1^) [26].

### 2.3. Light-activated heat generation in MWNT dispersions

The MWNT dispersion (0.02 mg/mL) and a control aqueous solution of 5% v/v Triton X-100 were exposed to continuous irradiation with a solar simulator (Oriel LCS-100) at 2 sun (200 mW/cm^2^) light density. Temperature was recorded every 3 min with a thermocouple (Hanna HI 935005 K-thermocouple thermometer). The thermocouple was immersed in the dispersion without blocking the path of the laser beam to avoid direct heating of the thermocouple by irradiation of the sunlight. Time-based temperature elevations were reported.

Overnight cultures (3 mL) of *P. aeruginosa* were grown in NB medium at 37 °C in a shaker incubator. The cells were washed twice by centrifugation at 5000 rpm for 5 min and resuspended in sterile phosphate-buffered saline (PBS). Next, 0.6 mL of bacterial suspensions (2 × 10^8^ CFU/mL) were mixed with 0.6 mL of dispersions of MWNTs (0.02 mg/mL) resulting in a dispersion with a MWNT concentration of 0.02 mg/mL and 2.5 % v/v Triton 100-X. Immediately after mixing, the dispersion was exposed to NIR laser irradiation with 1 W/cm^2^ at 808 nm for 15 min (STEMINC, SMM22808E1200) (Doral, FL USA). A control sample containing the same number of bacteria in 1.2 mL of water containing 2.5 % v/v Triton 100-X was also prepared and labeled as “cells only”. Both samples were irradiated with 808 nm NIR laser light for 15 min while recording the temperature. Following the light treatment, *P. aeruginosa* and *P. aeruginosa*-MWNT suspensions were centrifuged at 14,000 rpm for 5 min, and the supernatant was removed. The pellet containing the cells and MWNTs were fixed in 2.5 % glutaraldehyde in sterile PBS for 2 h at room temperature. The cells were rinsed twice with sterile PBS followed by dehydration through a series of increasing ethanol concentrations. Pellets were transferred onto aluminum SEM stubs and air dried. Secondary electron images of samples were obtained with a Zeiss Leo SUPRA 35 scanning electron microscope.

### 2.4. Preparation of PU-MWNT coatings

Three grams of aqueous PU dispersion with a solid content of 32% was thoroughly mixed with 1.2 mL and 6 mL of 0.017 mg/mL MWNT dispersions under overhead agitation and allowed to mix for 1 h, resulting in 0.002 wt.% and 0.01 wt.% MWNTs in solid PU, respectively. PU-MWNT dispersions were cast onto Petri dishes and dried overnight at room temperature followed by drying overnight in an oven at 50 °C to evaporate the water content and produce self-standing nanocomposite films. A PU film prepared by the same procedure without adding the MWNT dispersion was used as a control. Each film was cut into 1 × 1 cm pieces for further experiments.

### 2.5. Light-activated heat generation in MWNT-PU coatings

K type cable thermocouple was fixed in place between a Teflon plate and a piece of film (1 × 1 cm) for each sample. PU and MWNT-PU films were exposed to continuous irradiation from a solar simulator (Oriel LCS-100) at 2 sun (200 mW/cm^2^). The temperature was recorded every 3 min.

### 2.6. Light-activated biocidal properties of PU-MWNT coatings

After wiping PU and PU-MWNT film surfaces with ethanol, they were incubated with 2 mL of *P. aeruginosa* suspension containing 2 × 10^8^ CFU/mL in a 12-well plate overnight at 37 °C without shaking. The culture medium with nonadherent bacteria was removed, and the films were gently washed twice with sterile PBS. The films were stained using LIVE/DEAD BacLight kit and incubated for 10 min in the dark at room temperature. The excess staining solution was rinsed with PBS. The films were mounted onto coverslips and examined with a Carl Zeiss LSM 710 laser scanning confocal microscope equipped with a Plan-Apochromat 63×/1.40 oil objective before and after exposure to NIR laser light at 1 W/cm^2^ density for 15 min. Reported images are 3-D renderings of Z-stacks created using the Zen 2010 software.

To quantitatively determine the number of viable bacteria on the coated surfaces, another two sets of coated surfaces were incubated with 2 mL of *P. aeruginosa* suspension containing 2 × 10^8^ CFU/mL in a 12-well plate overnight at 37 °C. While one set of surfaces were kept in dark, the other set of surfaces were irradiated with NIR laser light at 1 W/cm^2^ density for 15 min. All surfaces (the ones that were kept in dark and the ones that were irradiated) were transferred into 5 mL sterile PBS and sonicated in a bath sonicator, so that the surface attached bacteria were transferred into the buffer. The resulting suspensions of bacteria were serially diluted and plated onto Agar plates. Agar plates were incubated overnight at 37 °C and colonies were counted. The number of viable bacteria was reported as log_10_ (CFU/cm^2^) of the mean and standard error obtained from triplicates.

### 2.7. Light-activated self-healing properties of PU-MWNT nanocomposites

PU dispersion and PU-MWNT dispersion containing 0.01 wt.% MWNTs were cast on petri dishes and dried to remove water as detailed above and 0.6 mm thick coatings were obtained. Onto each coating, a 1-cm wide scratch was inserted using a scalpel. Coatings were irradiated with a solar simulator at 2 sunlight density for 12 min and photographed every 4 min.

PU-MWNT films cast on the petri dishes were investigated by thermomechanical analysis (TMA) using a Mettler Toledo TMA/SDTA841e equipped with a ball point probe. Samples with a height of about 0.5 mm were cut from the bulk material and placed between two quartz glass disks. The TMA measurements were recorded at a heating rate of 10 K/min using a force of 0.025 N.

## 3. Results and discussion

### 3.1. Light-activated heat generation in MWNT dispersions

**Figure 1:**
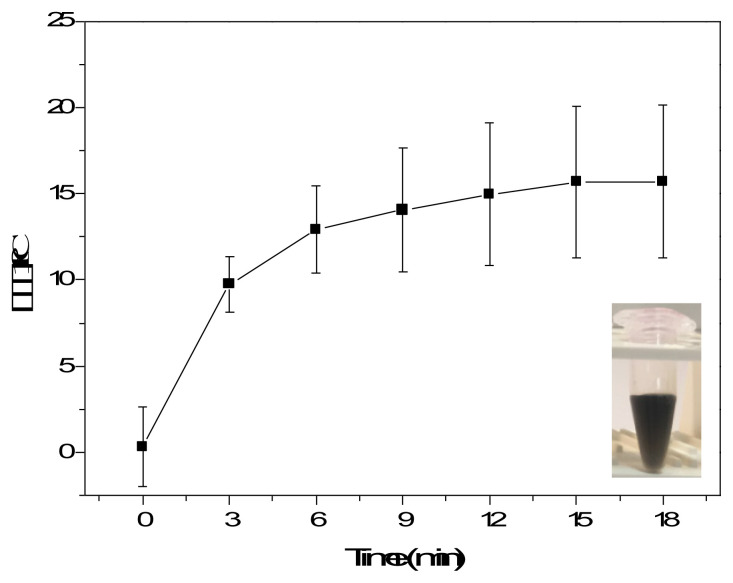
Temperature elevations in 0.02-mg/mL aqueous MWNT dispersion under sunlight at 2 sunlight density.

In order to impart waterborne PU with photothermal properties, aqueous PU dispersions were blended with MWNTs, which have strong light-to-heat conversion properties. The photothermal properties of aqueous dispersions of MWNTs were evaluated under a solar simulator providing sunlight at 2 sunlight density. The aqueous MWNT dispersion at 0.02 mg/mL reached temperatures 15 °C higher than water under sunlight irradiation for 18 min (Figure 1), demonstrating the photothermal conversion properties of the MWNTs and that they can be utilized as photothermal fillers to turn waterborne PU into a photothermal matrix.

**Figure 2:**
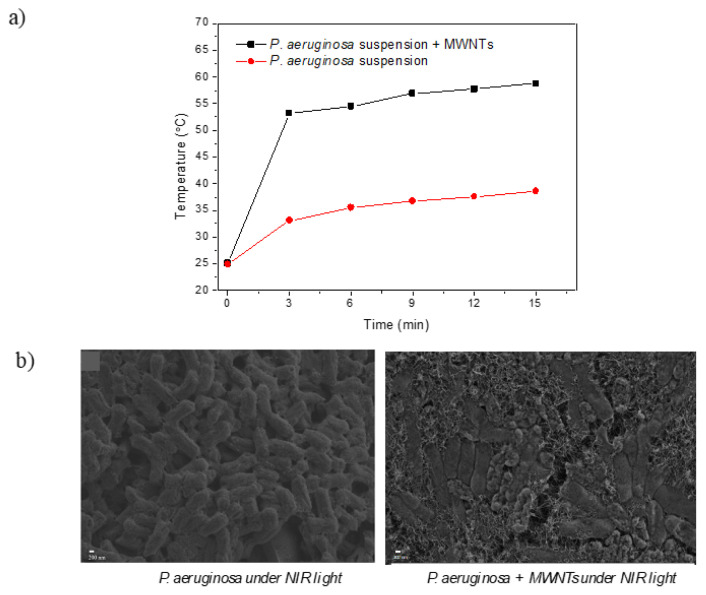
a) Time-temperature profiles of *P. aeruginosa* suspensions in the absence and presence of MWNTs under 808 nm laser irradiation at 1 W/cm^2^ light density, b) SEM images of *P. aeruginosa* cells, and *P. aeruginosa* cells treated with MWNTs after NIR irradiation for 15 min at 1 W/cm^2^ light density.

In order to evaluate whether the NIR light-activated temperature elevations can be utilized to physically disrupt and kill pathogenic cells, aqueous MWNT dispersions were mixed with aqueous suspensions of *P. aeruginosa*. The time-temperature profiles of the *P. aeruginosa* suspensions with and without MWNTs were constructed under NIR irradiation from an 808 nm laser light source. While the *P. aeruginosa* suspensions were mildly heated to 35 °C under NIR irradiation for 15 min, in the presence of the MWNTs, the same suspension was heated to 60 °C under the same conditions (Figure 2a). The MWNTs allowed sunlight-activated heating of the bacteria suspension to temperatures, which are lethal to bacteria. The effect of the light-activated temperature elevations on the *P. aeruginosa* was demonstrated in Figure 2b. The bacteria treated with MWNTs along with bacteria that were not treated with MWNTs were imaged with SEM after 15 min NIR light irradiation. While the physical appearances of the *P. aeruginosa* cells were not affected from the NIR irradiation, in the presence of MWNTs, their morphology was totally disrupted, and the cells lost their integrity. This demonstrated that the temperature elevations originating from the light-activation of MWNTs have caused hyperthermia resulting in killing of the bacteria, consistent with data reported in the literature about the hyperthermia-based killing effect of carbon nanotubes [27–29].

### 3.2. Photothermal properties of PU-MWNT coatings

**Figure 3:**
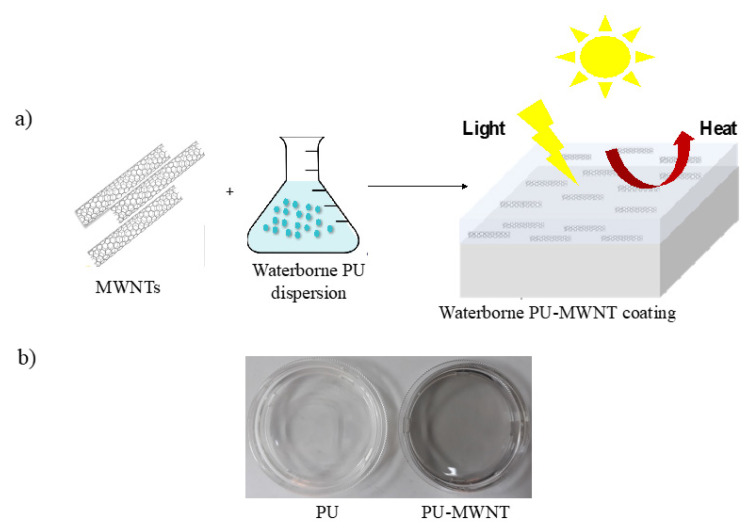
a) Schematic representation of the preparation of the photothermal waterborne PU-MWNT coatings, b) photographs of PU and PU-MWNT coatings on petri dishes.

Dispersions composed of 32 wt.% polyester-based PU and 68 wt.% water were mixed with MWNTs at different weight ratios under strong agitation from which self-standing films were cast through evaporation of the water content (Figure 3a). PU-MWNT films appeared to have a tint of black confirming the incorporation of the black MWNT powder into the transparent PU and its homogeneous distribution within the PU matrix (Figure 3b).

**Figure 4:**
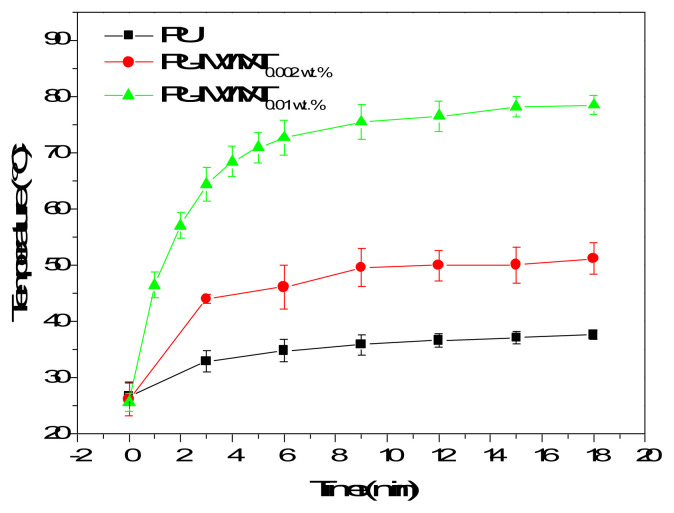
Time temperature profiles of PU, PU-MWNT_0.002 wt.%_, and PU-MWNT_0.01 wt.%_ films under sunlight irradiation at 2 sun.

The photothermal properties of the PU-MWNT nanocomposite films were evaluated by constructing their timetemperature profiles under sunlight irradiation. PU-MWNT nanocomposite films containing 0.01 wt. % MWNTs were heated to 80 °C under 18 min of sunlight irradiation. Under the same conditions, the neat waterborne PU film did not heat up significantly and its temperature reached only 35 °C (Figure 4). The MWNTs homogeneously distributed in the PU matrix absorbed the sunlight and converted it to heat resulting in remote sunlight-activated temperature elevations in the PU-MWNT nanocomposite films. The fact that the degree of the temperature elevations increased with increasing weight ratio of the MWNTs in the nanocomposite films further confirmed that the sunlight-activated temperature elevations occurred due to the presence of the photothermal MWNTs.

**Figure 5:**
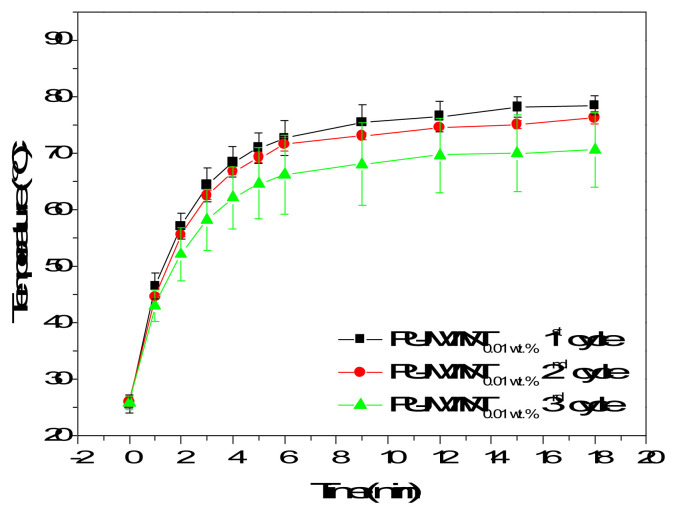
Time temperature profiles of PU-MWNT_0.01 wt.%_ films exposed to three consecutive sunlight on-off cycles at 2 sunlight density.

The photothermal stability of the PU-MWNT nanocomposite films was tested in three consecutive sunlight activation/cooling cycles to understand whether the light-activated heating of the films can be preserved when being reused. When the sunlight source was switched off after 18 min of irradiation and PU-MWNT_0.01 wt.%_ films cooled down to room temperature, they were able to heat up to the same temperature when they were irradiated with sunlight again (Figure 5). The photothermal effect originating from the MWNTs in the nanocomposite remained stable and the nanocomposite films were demonstrated to be photothermally activated in multiple cycles. This result showed that the PU-MWNT photothermal films can be reused in applications requiring multiple irradiation cycles.

### 3.3. Antibacterial properties of PU-MWNT coatings

**Figure 6:**
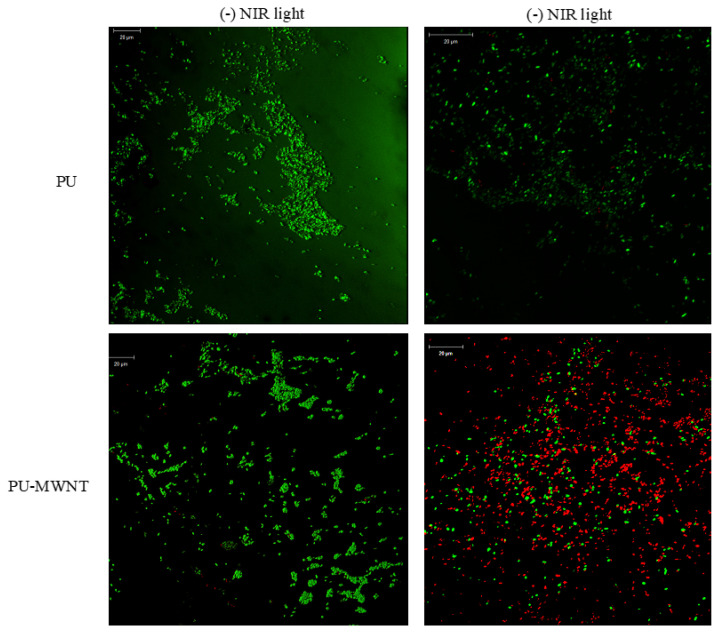
*P. aeruginosa* attached to PU- and PU-MWNT_0.01 wt.%_-coated surfaces visualized before and after 15 min NIR irradiation at 1 W/cm^2^ light density.

The potential of the PU-MWNT nanocomposites as light-activated antibacterial coatings was studied. Films were incubated with suspensions of *P. aeruginosa* to allow bacterial attachment on surfaces and the surfaces were irradiated with NIR light to evaluate whether the surface-attached bacteria can be killed by light activation. Bacteria-attached surfaces were labeled with live/dead indicator dyes and visualized with confocal microscopy before and after NIR light irradiation for 15 min (Figure 6). While bacteria attached to the neat PU-coated surface remained alive after NIR irradiation, bacteria on the PUMWNT _0.01 wt.%_-coated surfaces were killed under the same conditions, demonstrating the NIR-light-activated antibacterial properties of the PU-MWNT nanocomposites.

**Figure 7:**
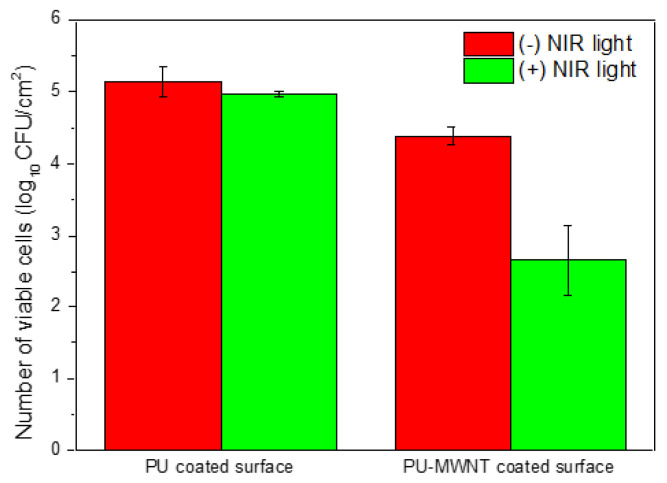
Viability of *P. aeruginosa* attached to PU- and PU-MWNT_0.01 wt.%_- coated surfaces before and after 15 min NIR irradiation at 1 W/cm^2^ light density.

The number of viable bacteria on the PU and PU-MWNT_0.01 wt.%_ coated surfaces before and after irradiation with NIR laser light for 15 min was also calculated (Figure 7). While bacteria attached to neat PU coated surfaces were not killed upon NIR laser light irradiation, the number of bacteria attached to the PU-MWNT_0.01 wt.%_ surface was reduced by approximately 2 log (99%) when the surface was exposed to NIR light. Upon NIR light irradiation, the MWNTs in the nanocomposites were heated to temperatures which cause hyperthermia in the bacteria cells and the bacteria were physically disrupted via temperature elevations. While some carbon nanomaterials such as fullerenes or single-walled carbon nanotubes are known to present a photodynamic effect and act as photosensitizers, MWNTs have been demonstrated not to generate radicals under NIR [30, 31]. This information further confirms that the antibacterial activity originated from the light-activated heating of the MWNTs. Thus, the waterborne PU-MWNT nanocomposites have a strong potential as coatings that allow light-activated remote sterilization of surfaces.

### 3.4. Self-healing properties of PU-MWNT coatings

**Figure 8:**
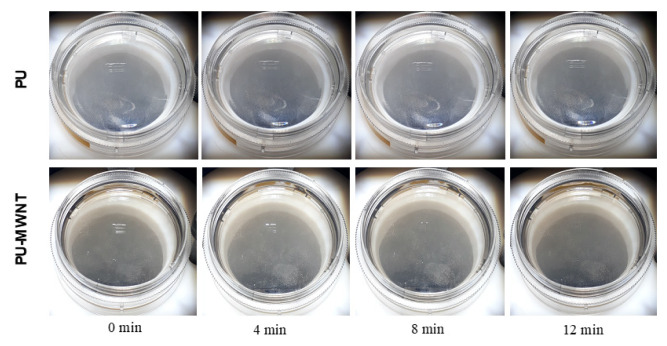
Self-healing of a scratch made on PU and PU-MWNT_0.01 wt.%_ coatings under sunlight at 2 sunlight density.

**Figure 9:**
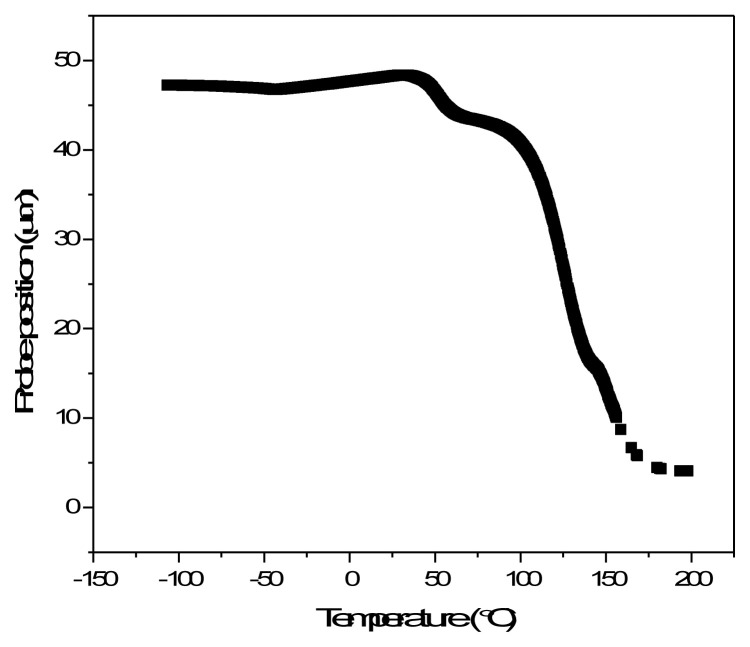
TMA of the PU-MWNT_0.01 wt.%_ nanocomposite.

Photothermal initiation of self-healing in materials has been demonstrated before [18, 32]. Nanofillers with photothermal properties were incorporated into polymer matrices resulting in nanocomposites with light-activated self-healing properties. As another potential application of PU-MWNT coatings, their sunlight-activated self-healing properties were studied. Petri dishes were coated with 0.6 mm PU and PU-MWNT nanocomposites and 1 cm scratches were made on each of the coatings. Both coatings with scratches were irradiated with sunlight at 2 sunlight density and the disappearance of the scratch was monitored (Figure 8). While the scratch on the PU coating remained unchanged during 12 min of sunlight irradiation, the scratch on the PU-MWNT coating slowly disappeared under sunlight and the polymer was totally healed after 8 min of irradiation. Figure 9 demonstrates the TMA of the PU-MWNT nanocomposites. In addition to the glass transition of the soft segment of the polyurethane at −50 °C, the graph clearly demonstrates the softening of the polymer as it was heated above room temperature. Most probably, the sunlight-activated heating of the PU-MWNT coating allowed the softening of the polymer through dissociation of the H-bonds of the hard segment and the polymer chains became more mobile [33], resulting in the healing of the scratch. This result demonstrated that PU-MWNT-coated surfaces can be utilized for remote self-healing of surfaces via sunlight activation.

## 4. Conclusion

Waterborne PU was turned into photothermal coating materials by the incorporation of MWNTs, which were shown to present light-to-heat conversion properties. PU-MWNT nanocomposites were demonstrated to present significant temperature elevations when irradiated with NIR light, which can be utilized in a variety of applications. NIR light from an 808 nm laser or sunlight from a solar simulator can remotely heat the MWNT-PU coatings to high temperatures at which bacteria can be killed via hyperthermia or polymer self-healing can occur. Waterborne polyurethanes, which are environmentally friendly, versatile, tunable polymers being used in various industrial applications, were imparted photothermal character and resulting coating materials were demonstrated to present strong light-to-heat conversion properties.
